# Longitudinal changes in objective sleep parameters during pregnancy

**DOI:** 10.1177/17455057231190952

**Published:** 2023-08-31

**Authors:** Yuqing Guo, Qi Xu, Nikil Dutt, Priscilla Kehoe, Annie Qu

**Affiliations:** 1 Sue & Bill Gross School of Nursing, University of California, Irvine, Irvine, CA, USA; 2 Department of Statistics, Donald Bren School of Information & Computer Sciences, University of California, Irvine, Irvine, CA, USA; 3 Donald Bren School of Information & Computer Sciences, University of California, Irvine, Irvine, CA, USA

**Keywords:** longitudinal changes, objective sleep parameters, pregnancy

## Abstract

**Background:**

Sleep disturbances are associated with adverse perinatal outcomes. Thus, it is necessary to understand the continuous patterns of sleep during pregnancy and how moderators such as maternal age and pre-pregnancy body mass index impact sleep.

**Objective:**

This study aimed to examine the continuous changes in sleep parameters objectively (i.e. sleep stages, total sleep time, and awake time) in pregnant women and to describe the impact of maternal age and/or pre-pregnancy body mass index as moderators of these objective sleep parameters.

**Design:**

This was a longitudinal observational design.

**Methods:**

Seventeen women with a singleton pregnancy participated in this study. Mixed model repeated measures were used to describe weekly patterns, while aggregated changes describe these three pregnancy periods (10–19, 20–29, and 30–39 gestational weeks).

**Results:**

For the weekly patterns, we found significantly decreased deep (1.26 ± 0.18 min/week, p < 0.001), light (0.72 ± 0.37 min/week, p = 0.05), and total sleep time (1.56 ± 0.47 min/week, p < 0.001) as well as increased awake time (1.32 ± 0.34 min/week, p < 0.001). For the aggregated changes, we found similar patterns to weekly changes. Women (⩾30 years) had an even greater decrease in deep sleep (1.50 ± 0.22 min/week, p < 0.001) than those younger (0.84 ± 0.29 min/week, p = 0.04). Women who were both overweight/obese and ⩾30 years experienced an increase in rapid eye movement sleep (0.84 ± 0.31 min/week, p = 0.008), but those of normal weight (<30 years) did not.

**Conclusion:**

This study appears to be the first to describe continuous changes in sleep parameters during pregnancy at home. Our study provides preliminary evidence that sleep parameters could be potential non-invasive physiological markers predicting perinatal outcomes.

## Introduction

Hormonal changes and physical discomfort influence pregnant women’s sleep.^1^ Approximately 46% of pregnant women experience self-reported poor sleep quality, and one-third have insufficient sleep.^1,2^ Sleep disturbances (i.e. disturbed sleep quantity and quality) are associated with adverse perinatal outcomes such as gestational diabetes, hypertension, preterm birth, and postpartum depression.^3–6^ Sleep stages (objective parameters of sleep quantity and quality) capture important indicators of health.^7^ Deep sleep approximately accounts for 20% of total sleep and is characterized by a significant restorative effect during which an individual’s immune functions are enhanced.^7^ Rapid eye movement (REM) sleep is characterized by vivid dreams and enhanced memory consolidation, accounting for 25% of total sleep.^7–9^ Light sleep makes up 55% of total sleep which includes 5% of the transitional phase from awake to sleep and 50% intermediate phase of sleep.^7,8^ For example, increased growth hormone and decreased cortisol are associated with deep sleep.^10^ Despite emerging literature on sleep during pregnancy and its predictive significance, there is a lack of understanding of the magnitude of continuous changes in sleep parameters (particularly sleep stages) over pregnancy. This gap in the research is primarily due to the methods of assessing sleep. Specifically, self-reported questionnaires (e.g. Pittsburgh Sleep Quality Index: PSQI) have been utilized in most of the existing literature^2^; however, these subjective ratings of sleep compared with objective measurements of sleep vary from poor to low associations.^11,12^

More recently, studies are using actigraphy to objectively measure sleep among perinatal women. Specifically, actigraphy uses a motor sensor (accelerometer) to derive sleep and wake patterns,^13^ such as total sleep duration and awake time, but does not assess stages of sleep including deep, REM, and light sleep.^4,14,15^ Of these studies using actigraphy during pregnancy, a few evaluated sleep characteristics for 3–7 days in one trimester,^4,14,15^ and one recent research monitored sleep characteristics for a selected 14 consequent days of each trimester.^16^ In addition, a Garmin watch was utilized to continuously track total sleep duration and awake time in nulliparous women throughout 6-month pregnancy and 1-month postpartum.^17^ The accumulative evidence shows that sleep declines in terms of quantity and quality during pregnancy, being more pronounced in the third trimester.^2,17^

In addition, a few studies utilized polysomnography (PSG) that includes sleep electroencephalogram (EEG) to measure changes in sleep stages for 1 or 2 days at various points during pregnancy.^18–20^ PSG is considered a gold standard for sleep assessment often done in the laboratory or clinical setting that combines EEG with a recording of chest/abdomen movement via plethysmography, as well as oxygen saturation using pulse oximetry. Recent PSG research shows that greater deep sleep was associated with lower inflammatory biomarkers, while higher light sleep was associated with increased inflammatory biomarkers in pregnant women.^21^

Furthermore, sleep varies based on several factors such as maternal age and body mass index (BMI). Pregnant women over 30 years reported shorter sleep duration compared with those under 30 years.^22^ Overweight or obese pregnant women experienced greater sleep disturbances indicated by self-reported PSQI.^23^ However, there is little knowledge on how age and/or BMI impact objective sleep parameters in pregnant women.

This study addresses these gaps by applying digital health using an Oura smart ring to daily monitor the objective sleep parameters at night across the entire pregnancy at home. The aims were to (1) examine the continuous changes in sleep parameters objectively (i.e. sleep stages, total sleep time, and awake time) in pregnant women, and (2) describe the impact of maternal age and/or pre-pregnancy BMI as moderators of these objective sleep parameters.

## Methods

### Study design and sampling

This study used a longitudinal prospective observation design, a part of the parent project with the purpose of understanding the feasibility of using digital health to examine biopsychosocial changes in underserved pregnant women.

All research procedures were conducted in accordance with the required ethical standards. As recruitment began, the COVID-19 Stay-At-Home Restriction came into effect, influencing interactions with subjects, mainly requiring virtual contact. Convenience sampling was used to recruit participants. We shared the study flyer with community partners engaging in work with underserved perinatal women in Orange County, California, resulting in mostly Hispanic women being recruited. Inclusion criteria were pregnant women aged 18–40 years, having a healthy singleton pregnancy at enrollment, and access to a smartphone. The exclusion criteria included being older than 40 years, having medical complications, and not possessing a smartphone. The research coordinator (RC) screened potential subjects, consented, and enrolled eligible participants. The subjects were informed about the goals of the research, potential benefits, and risks. The verbal informed consent was granted by UCI IRB. The RC documented consent in the research protocol, and all the participants received an IRB-approved Study Information Sheet as their record ensuring that they could contact the research team and/or IRB with any inquiry. We instructed the women to wear the smart ring as much as possible throughout their pregnancy, particularly at nighttime. There were 53 potential subjects screened. Twenty subjects were eligible to participate and received informed consent; two dropped out early due to family circumstances. The sample size was determined by the parent project being a feasibility study to pilot using technology to understand objective biopsychosocial changes in underserved pregnant women. The recommended average sample size of a feasibility study ranges from 20 to 35 subjects.^24,25^ One participant only had 7-day smart ring data due to pregnancy complications. Thus, 17 subjects’ data were used. There were 1100 days of participation in the study with 73 missing days and 4 outlier days which led to 1023 days of sleep data for analyses. In addition, we followed the STrengthening the Reporting of OBservational studies in Epidemiology (STROBE) Guidelines to organize the “Methods” section when preparing the article.

### Data collection procedure

All research protocols were tailored to the COVID-19 Stay-At-Home Restriction. Specifically, the RC collected the self-reported demographic data through REDCap (a secure data collection platform), including maternal age, education, ethnicity, parity, and pre-pregnancy BMI. The Oura ring is a waterproof multi-sensor wearable device detecting physiological signals using an optical pulse waveform from a participant’s finger. The ring transfers data to an App installed on the participants’ smartphone automatically via Bluetooth (Oulu, Finland). Each consented participant chose a ring size that she could wear comfortably on her finger, and then the ring was shipped to her. All participants were provided with standardized instructions on installing and using the ring. All communications between the study team and participants were conducted virtually, from October 2020 to 2021. Each subject received a $200 gift card as compensation for their participation in the study.

### Measures

Previously, Oura ring was validated against PSG, showing that it can detect wake–sleep patterns and sleep stages with acceptable accuracy, sensitivity, and specificity ranging from 79% to 96%.^26^ We used the smart ring to measure daily nocturnal sleep data (napping is not assessed) at home. Nocturnal sleep parameters are detected and calculated by using the combination of nighttime movement, resting heart rate/heart rate variability, and pulse wave variability amplitude collected from photoplethysmography, negative temperature coefficient thermistor, and a 3D accelerometer as well as leveraging machine learning methods.^26^ Oura ring calculates sleep stages every 30 s throughout the night and provides the summary night data of each sleep parameter. We instructed the women to wear the smart ring for 24 h throughout the pregnancy, most importantly at night. The ring synchronized with the participant’s mobile app that showed the summary of sleep parameters. Sleep stages comprise the hours of deep, REM, and light sleep. Total sleep time refers to the total amount of sleep that is registered during the time in bed. Awake time refers to the total time of wakefulness occurring after sleep onset.^27^ We obtained the Oura company’s permission to use the data for analyses in this study.

### Statistical analyses

We used daily average sleep parameters obtained from the smart ring for our 17 subjects. First, we pre-processed the collected daily data to manage outliers and checked normality. Second, we developed sleep data over longitudinal time by calculating subject’s weekly average sleep parameters. We used mixed model repeated measures to detect weekly changes in these sleep parameters over gestation. Third, these parameters were aggregated for the 17 subjects over the three periods (10–19, 20–29, and 30–39 gestational weeks [GWs]) closely based on characteristic changes during pregnancy described by the American College of Obstetricians and Gynecologists^28^; we also used mixed model repeated measures to compare the aggregated changes in the sleep parameters. Finally, we included age and/or pre-pregnancy BMI into these models to explore their moderating effects on these sleep parameters. Specifically, we grouped the subjects by maternal age ⩾ 30 years (n = 7) and age < 30 years (n = 10). Similarly, subjects were categorized as normal-weight group (pre-pregnancy BMI ⩽ 25.0, n = 7) and overweight/obese group (pre-pregnancy BMI > 25.0, n = 10). Three interaction terms were created and added to the models including GW and age, GW and pre-pregnancy BMI, and GW and age/pre-pregnancy BMI. All the above pre-processing and statistical analyses were performed in R (a software for statistical computing and graphics, R-4.2.0 for Mac) with p ⩽ 0.05.

## Results

The baseline results showed that most women (15, 88%) self-identified as Hispanic. The average age of participants was 27.8 years (SD = 4.48), and the average gestation at the enrollment was 19.78 (SD = 5.88) weeks. Six (35%) women were first-time mothers. More than half of the women (10, 59%) were overweight or obese. Two (11%) women had high school diplomas, six (35%) had some college education or associate degree, and nine (54%) had a bachelor’s or master’s degree.

**Figure 1.**
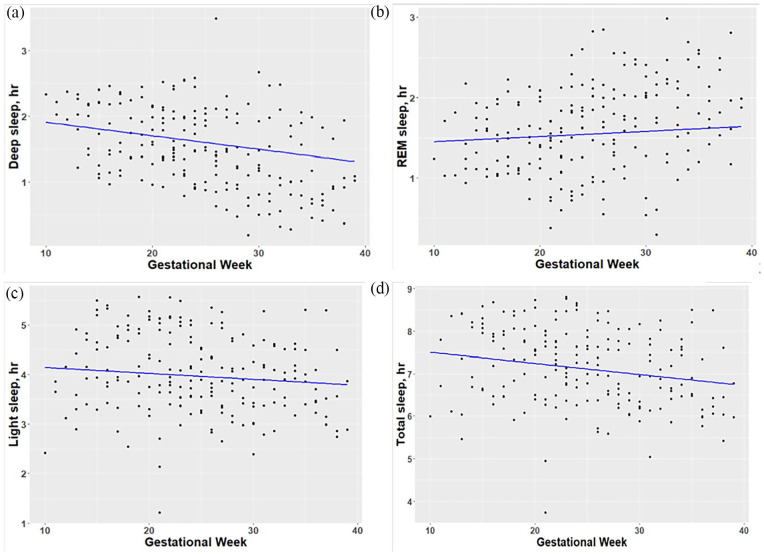
Weekly changes in sleep parameters by gestational weeks: (a) deep sleep, (b) REM sleep, (c) light sleep, and (d) total sleep time.

Across all subjects using linear mixed models to describe the weekly sleep patterns, we found that there were significant decreases in deep sleep (1.26 ± 0.18 min/week, p < 0.001), light sleep (0.72 ± 0.37 min/week, p = 0.05), and total sleep time (1.56 ± 0.49 min/week, p < 0.001), as well as a significant increase in awake time (1.32 ± 0.34 min/week, p < 0.001). However, for REM sleep, there was little change (0.39 ± 0.23 min/week, p = 0.09). In other words, from early to late pregnancy (10–39 GWs), we estimated that there were approximate reductions in these sleep parameters as follows: deep (36.54 min), light (20.88 min), and total sleep (45.24 min), whereas there was an increase in awake time (38.28 min). Figure 1(a)–(d) scatterplots demonstrate weekly changes in sleep stages (deep, REM, and light sleep) and total sleep time over pregnancy.

Table 1 shows patterns of aggregated sleep parameters by three pregnancy periods. These results were similar to weekly changes as above in that deep, light, and total sleep were significantly decreased while the awake time significantly increased, and REM had little change across these periods.

**Table 1.** Patterns of aggregated sleep parameters over pregnancy.

Pregnancy period (gestational weeks)	(10–19) Mean (SD)	(20–29) Mean (SD)	(30–39) Mean (SD)	p value
Deep^a^ (hr)	1.80 (0.47)	1.54 (0.54)	1.17 (0.61)	<0.0001
REM^b^ (hr)	1.45 (0.39)	1.61 (0.54)	1.83 (0.59)	0.6123
Light^c^ (hr)	4.19 (0.81)	4.06 (0.82)	3.80 (0.68)	0.0309
Total^d^ (hr)	7.43 (0.88)	7.21 (0.92)	6.81 (0.82)	0.0015
Awake^e^ (hr)	1.23 (0.42)	1.19 (0.53)	1.63 (0.77)	0.0003

SD: standard deviation.

a Deep sleep.

b Rapid eye movement sleep.

c Light sleep.

d Total sleep.

e Awake time.

**Figure 2.**
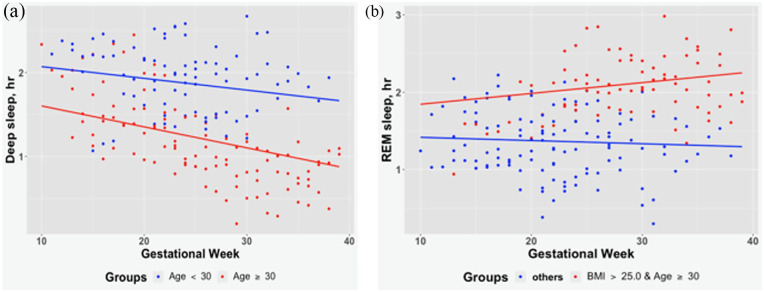
Moderators for deep sleep and REM sleep: (a) Maternal age as a moderator for deep sleep. (b) Maternal age and pre-pregnancy BMI as moderators for REM sleep.

We found that maternal age moderated deep sleep, but not other sleep parameters. Specifically, using subgroup analyses, Figure 2(a) shows that pregnant women (⩾30 years) had a greater decrease in deep sleep over time compared to women under 30 years respectively (1.50 ± 0.22 min/week, p < 0.001 versus 0.84 ± 0.29 min/week, p = 0.04). Pre-pregnancy BMI alone did not significantly moderate any sleep parameters. However, both maternal age and pre-pregnancy BMI together moderated REM sleep (p = 0.02), but not other sleep parameters. In addition, Figure 2(b) demonstrates that those women who were both aged ⩾30 years and overweight/obese experienced a significant increase in REM over time, while their counterparts did not (0.84 ± 0.31 min/week, p = 0.008 versus −0.24 ± 0.34 min/week, p = 0.46).

## Discussion

### Weekly patterns of sleep parameters in pregnant women

To the best of our knowledge, this study was the first to describe the continuous sleep patterns over pregnancy using a validated Oura ring in a home setting. We found significant changes in sleep parameters in terms of decreased deep and total sleep time as well as increased awake time across GWs. These longitudinal results with a smart ring align with those using 1–2 days of polysomnography at different trimesters in healthy women.^19,20^ We extended the existing literature by describing the magnitude of these changes and establishing the actual weekly change rates in sleep parameters in healthy women over pregnancy. Notably, we found that light sleep significantly decreased, thus contributing to the mixed results in previous studies. One used PSG to examine each woman for two nights during 18–23 GWs and two nights during 32–35 weeks and observed a decline in light sleep.^18^ However, another similarly executed study using PSG showed an increase in light sleep,^19^ but that study only included pregnant women who were at risk for sleep-disordered breathing. For REM, we found no change across GWs,^18^ but some found a decrease in REM.^17,18^ However, we did find an increase in REM sleep that was associated with the moderating effects of maternal age and pre-pregnancy BMI together that were not examined by the prior studies.

### Maternal age and/or pre-pregnancy BMI as moderators on sleep parameters

Our study is the first to investigate how moderators impacted sleep parameters. Pregnant women being 30 years of age or over had a greater reduction in deep sleep by 1.50 min/week compared with the reduction of 0.84 min with their younger counterparts. Our study found a moderating role of age on deep sleep in pregnancy as did previous research with older but non-pregnant women who had shorter duration of deep sleep compared with those younger.^29^ We also found that pregnant women being 30 or over as well as overweight/obese had increased REM sleep by 0.84 min/week, results not found in younger normal-weight women. Interestingly, it appears that non-pregnant overweight/obese individuals without sleep disordered breathing had significantly higher REM than normal-weight counterparts.^30^ More studies are needed to further investigate the pattern of REM over pregnancy and the factors that may modify it.

### Implications of sleep parameters

Emerging evidence shows that sleep disturbances are a risk factor for adverse pregnancy outcomes through dysregulated inflammatory pathways.^3,10,31^ Although pregnancy is characterized by a balance of immunologic changes, excessive increased pro-inflammatory markers can lead to adverse pregnancy outcomes.^31,32^ A recent study demonstrated that pregnant women who experienced longer duration of deep sleep had lower evening and morning C-reactive protein (CRP).^21^ Conversely having higher CRP may be a risk factor since increased CRP has been shown to be associated with gestational diabetes and preeclampsia.^33,34^ Furthermore, decreased deep sleep was also associated with other pro-inflammatory responses such as increased tumor necrosis factor alpha (TNF-α).^35^ Notably, higher TNF-α was found in women who experienced recurrent spontaneous pregnancy loss compared with those with normal pregnancies.^36^ We showed that pregnant women 30 or over had a tendency for lesser deep sleep perhaps putting them at risk for higher pro-inflammatory levels.

Increased light and REM sleep was associated with higher levels of interleukin 6 (IL-6).^21,37^ It is noted that elevated IL-6 has been found to be associated with an early labor and delivery process.^38^ Besides infections, the high pro-inflammatory responses to sleep disturbance and/or stress may be one pathway to preterm births.^10^ In addition, pre-pregnancy BMI was associated with higher IL-6 across pregnancy.^32^ We found that overweight/obese pregnant women 30 or over had experienced longer REM sleep time probably exposing them to the vulnerability of elevated inflammatory responses. Our study suggests that monitoring sleep parameters could be a potential non-intrusive method to assess risk factors such as sleep disturbances for adverse pregnancy outcomes. More studies are needed to differentiate the amount of sleep changes that are within normal limits as opposed to changes that negatively impact inflammatory pathways.

### Limitations

We recognize a few limitations. First, emerging evidence shows that parity influences some sleep parameters. In a study using PSG, nulliparous mothers experienced significantly lower sleep efficiency compared with multiparous mothers; but such a difference was not found in deep and REM sleep.^20^ Due to a small number of first-time mothers in our study, parity was thus not included in the linear mixed models. Second, overweight/obese pregnant women are at risk for sleep breathing disorders, particularly obstructive sleep apnea.^39^ However, the diagnoses of sleep disorders were not available in this study. In addition, weight gain was not collected in this study. It is important to investigate how weight gain would impact sleep parameters in future studies. Third, there is mixed evidence that wearables may prompt behavior change.^40,41^ All women who participated in this study had access to their sleep data on their smartphone. This could be a potential bias on their sleep patterns. Therefore, caution is needed to interpret the results. Finally, given that this study was designed to test the feasibility of using the smart ring to collect objective sleep parameters, the power analysis was not conducted. Thus, the small sample size could be another bias. Furthermore, most women being Hispanic limits the generalization of results. Future studies with pregnant women are warranted to confirm our findings with a larger sample size including other ethnicities, and how parity and sleep disorders impact objective sleep parameters over pregnancy.

## Conclusion

The contribution of this study is the longitudinal and continuous monitoring of objective sleep parameters using a wearable device in the home setting among primarily Hispanic pregnant women in the United States. We identified sleep physiological patterns that were characterized by decreased deep, light, and total sleep time but increased awake time. Furthermore, our results suggest that maternal age and/or pre-pregnancy BMI are risk factors for changes in sleep patterns. Importantly, our study provides preliminary evidence that understanding of objective sleep characteristics over pregnancy (particularly sleep stages) could be potential biomarkers predicting perinatal outcomes.

## Supplemental Material

sj-docx-1-whe-10.1177_17455057231190952 – Supplemental material for Longitudinal changes in objective sleep parameters during pregnancy Click here for additional data file. Supplemental material, sj-docx-1-whe-10.1177_17455057231190952 for Longitudinal changes in objective sleep parameters during pregnancy by Yuqing Guo, Qi Xu, Nikil Dutt, Priscilla Kehoe and Annie Qu in Women’s Health

## Declarations

**Ethics approval and consent to participate:** Ethical approval for this study was obtained from the University of California Irvine (UCI) Office of Research Institutional Review Board (IRB) (HS# 2019-5363). The UCI IRB approved the verbal informed consent in that this study was considered as low risk for subjects. In other words, the trained research coordinator provided a detailed explanation of the study using an IRB-approved Study Information Sheet to all subjects. Subject consent for participation in the study was obtained during the recruitment process and was completed before the beginning of the study. All the consenting information was recorded in the research protocol.

**Consent for publication:** All participants gave consent for their data to be used in publications.

**Availability of data and materials:** The datasets used and/or analyzed during this study are available from the corresponding author on reasonable request.
